# Hybridization and Asymmetrical Introgression Between the Vulnerable Gray‐Headed Chickadee and a More Abundant Congener, the Boreal Chickadee: Implications for Conservation

**DOI:** 10.1002/ece3.71673

**Published:** 2025-06-26

**Authors:** Matthew R. Armstrong, Robert E. Wilson, James A. Johnson, Travis L. Booms, Callie F. Gesmundo, Zachary M. Pohlen, Paul B. Leonard, Sarah A. Sonsthagen

**Affiliations:** ^1^ School of Natural Resources University of Nebraska Lincoln Lincoln Nebraska USA; ^2^ Nebraska State Museum University of Nebraska Lincoln Lincoln Nebraska USA; ^3^ U.S. Fish and Wildlife Service Migratory Bird Management Anchorage Alaska USA; ^4^ Alaska Department of Fish and Game Fairbanks Alaska USA; ^5^ U.S. Fish and Wildlife Service‐Arctic National Wildlife Refuge Fairbanks Alaska USA; ^6^ U.S. Geological Survey‐Nebraska Cooperative Fish and Wildlife Research Unit, School of Natural Resources University of Nebraska Lincoln Lincoln Nebraska USA

**Keywords:** interspecific hybridization, introgression, museomics, *Poecile cinctus lathami*, *Poecile hudsonicus*

## Abstract

Hybridization is a common process among bird species that can precipitate a mix of positive or negative species outcomes. Particularly for rare populations, detrimental effects of hybridization on demographic growth rates and genetic integrity are of serious concern. In Alaska and a small region of northwestern Canada, the endemic subspecies of Gray‐headed Chickadee ( *Poecile cinctus lathami* ) has declined in recent decades from being locally common to being extremely rare. The more widespread Boreal Chickadee ( *P. hudsonicus* ) has become increasingly abundant in areas of sympatry. These changes in abundance may have led to hybridization between Gray‐headed Chickadees and Boreal Chickadees. We used a series of analyses to test for signatures of introgression at mitochondrial DNA and nuclear DNA using historical museum samples of both species collected between 1875 and 1979 as well as contemporary Boreal Chickadee samples. In addition, we modeled Gray‐headed Chickadee and Boreal Chickadee demographic histories to better understand patterns of effective population size changes and gene flow over time. Introgression of Gray‐headed Chickadee nuclear DNA was detected in contemporary and historical Boreal Chickadee populations, and two first‐generation hybrid backcrosses were observed in the historical Boreal Chickadee samples. Lack of mitochondrial DNA introgression or backcrossing into the Gray‐headed Chickadee historical samples may be an artifact of mate scarcity during the period before local abundances of Boreal Chickadee exceeded Gray‐headed Chickadees. Demographic modeling with nuclear loci estimated a low level of symmetric gene flow between Gray‐headed Chickadees and Boreal Chickadees since the time of divergence. Our study suggests that hybridization may be linked to Gray‐headed Chickadee declines and represents a case study of how museum collections can be used to infer introgression in a population too scarce to directly investigate.

## Graphical abstract

We assess genetic evidence of hybridization between gray‐headed chickadees and boreal chickadees using mitochondrial DNA and nuclear DNA sequencing of individuals from historical and contemporary time periods. We detected two early‐generation hybrids among the historical boreal chickadee samples and none within the contemporary boreal chickadee samples collected after gray‐headed chickadee abundances decreased.

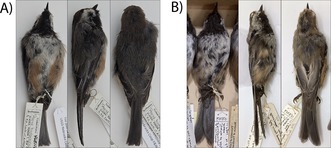

## 1 Introduction

Over the accelerated time span of anthropogenic‐mediated climate change, uneven shifts in the distribution of neighboring species may produce new or altered contact zones and provide opportunities for interspecific hybridization (Chunco 2014; Krosby et al. 2015; McQuillan and Rice 2015). Hybridization is dependent on species range overlap, and variable climate can lead to unstable species ranges. Rare species with limited distributions tend to occupy areas with localized climates that are susceptible to shrinking further with climate change (Ohlemüller et al. 2008). When neighboring species shift distributions in response to new conditions, rare species might be less able to shift in parallel and thus be particularly vulnerable to increased hybridization risk.

The effects of hybridization on populations can vary widely depending on factors such as fitness of hybrid offspring and the relative abundance of each species (Todesco et al. 2016). At the negative extreme, completely sterile hybrids would provide no reproductive potential while still competing for resources, leading to abundance declines. If hybrids only have partially reduced fitness, hybrid offspring may still decrease population growth rates of the less abundant species (i.e., demographic swamping; Wolf et al. 2001). Hybrids that backcross with one of the parental populations could introduce new alleles to those populations. Introgression of alleles may erode the genetic integrity of a parent population until pure parental genotypes are completely lost (i.e., genetic swamping; Todesco et al. 2016). Simulations suggest that pure parental genotypes of a rare species will become extinct even faster with greater initial disparities in population sizes (Epifanio and Philipp 2000), and loss of pure genotypes of the rare species could be further exacerbated if the more abundant species also experiences immigration from surrounding areas.

Positive effects of hybridization can occur as well (Hohenlohe et al. 2021), such as an increase in genetic diversity and heterozygosity, which may be beneficial to species affected by climate change. For example, snowshoe hares ( *Lepus americanus* ) in mild environments have benefited from adaptive introgression with jackrabbits ( *Lepus californicus* ) since the Last Glacial Maximum (Jones et al. 2018). Contact between these two species led to hybridization, and a subsequent genetic sweep at genes controlling pelage produced snowshoe hare populations with brown winter coats rather than the ancestral white coat phenotype. This study and others illustrate the challenge of predicting the outcome of hybridization linked to warming climates; possible results range from species extinction to rapid adaptation to those same climate change stressors (Chunco 2014).

Within birds (class Aves), hybridization has been documented in approximately 9%–19% of species; however, the true frequency of interspecific breeding is likely even higher given that not all hybrids are visually distinct (Grant and Grant 1992; Ottenburghs 2023). Evidence from the Tyranni suborder suggests that rates of hybridization over the past 21,000 years may be correlated with geographic proximity and rates of climate change (Singhal et al. 2021). In recent decadal time spans, geographic shifts have been observed in the location of hybrid zones for various avian species pairs (Buggs 2007). Rates of hybrid zone movement in some hybrid systems such as the Black‐capped Chickadee ( *Poecile atricapillus* ) and the Carolina Chickadee ( *P. carolinensis* ) appear to be associated with shifts in climate variables that determine one species' range limits (Taylor et al. 2014; Alexander et al. 2022).

**FIGURE 1:**
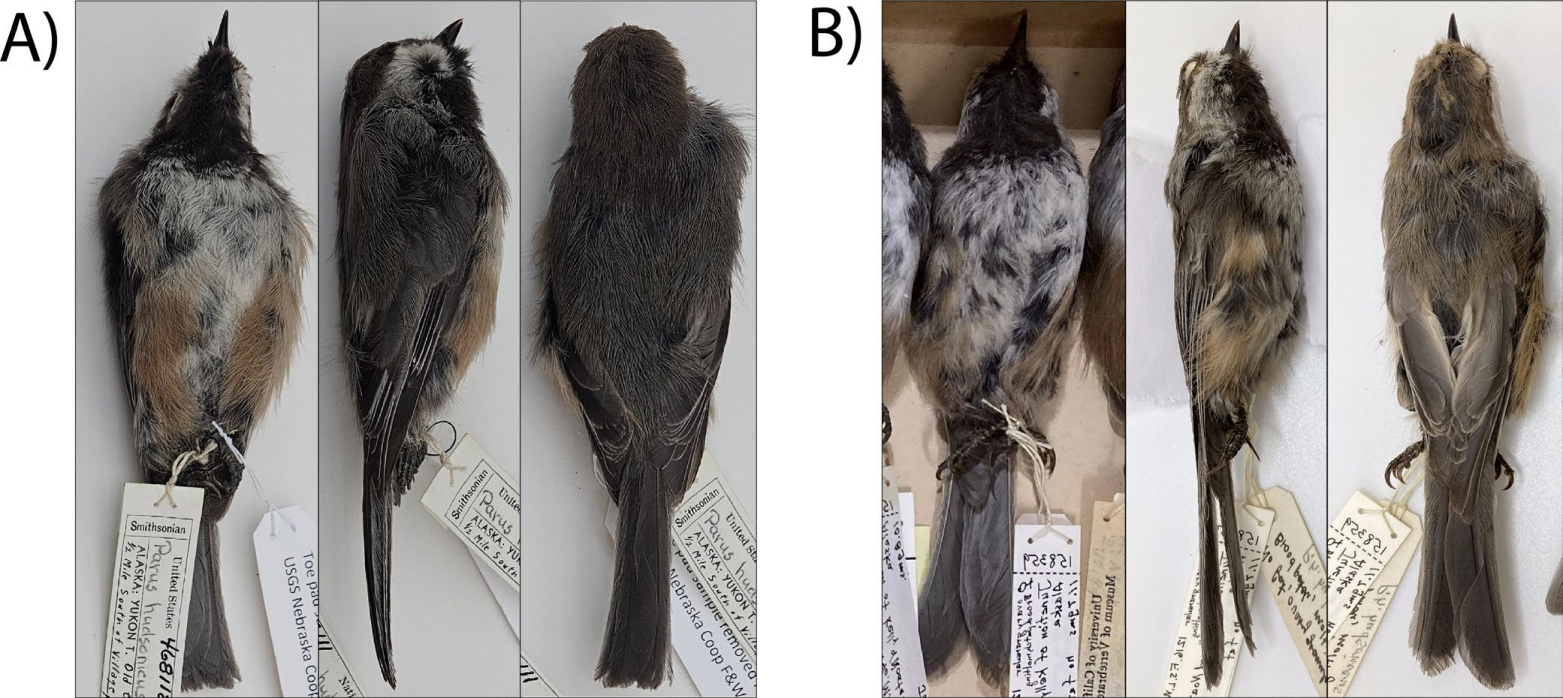
Photographs of Boreal Chickadee specimens (A) USNM: Bird: 468112, National Museum of Natural History, Smithsonian Institution and (B) MVZ: Bird: 158359, Museum of Vertebrate Zoology, University of California, Berkeley. Both specimens have genetic signatures associated with second‐generation hybrid backcrossing from Gray‐headed Chickadee. Photographs courtesy of Christopher Milensky and Elizabeth Beckman.

Researchers have identified hybridization between various pairs of chickadees, a clade of seven species in the *Poecile* genus (Curry 2005; Lait et al. 2024). Many studies focused on the impacts of hybridization on chickadee behavior and ecology investigated crosses between the Black‐capped Chickadee and either Carolina Chickadees or Mountain Chickadees ( *P. gambeli* ; e.g., McQuillan et al. 2018, Grabenstein et al. 2023). In contrast, little to none is known about potential hybridization between the widespread Boreal Chickadee ( *P. hudsonicus* ) and the remote, isolated North American populations of Gray‐headed Chickadee ( *P. cinctus* ; Figure 1). The Gray‐headed Chickadee, formerly known as the Siberian Tit, is primarily found throughout northern Eurasia. However, one of the four subspecies (*lathami*) is endemic to Alaska and a small portion of northwestern Canada. The Gray‐headed Chickadee is a small, non‐migratory cavity‐nester that primarily inhabits spruce (*Picea* spp.), willow (*Salix* spp.), and poplar (*Populus* spp.) habitats along rivers (Booms et al. 2020). Gray‐headed Chickadees are a species of conservation concern recognized by the U.S. Fish and Wildlife Service (“Birds of Conservation Concern 2021”) and an endangered species based on assessment by the Committee on the Status of Endangered Wildlife in Canada (“COSEWIC Wildlife Species Assessments May 2024”), but Gray‐headed Chickadees are not currently listed and protected under the U.S. Endangered Species Act or Canada's Species at Risk Act.

Records from 1864 until approximately 2011 indicated a low density of Gray‐headed Chickadees across their North America range, but anecdotes from several sites suggest they were locally common (Hines 1963; Booms et al. 2020). Observations at two specific sites (Brooks Foothills and Porcupine River drainage) have decreased in recent decades, whereas Boreal Chickadees have been noted in anecdotal reports and surveys at higher abundances than in the past (Booms et al. 2020). Booms et al. (2020) detected three Gray‐headed Chickadees on 400 km of line transect surveys conducted in suitable habitat during 2010–2017. Additionally, the last reliable observation of a Gray‐headed Chickadee occurred in 2017, suggesting that Gray‐headed Chickadees are extirpated from southwestern and Interior Alaska and Northwest Territories (Booms et al. 2020). The absence of known extant populations largely precludes an assessment of factors that might explain reduced populations, such as competition with other species over foraging or nesting sites, disease, or climate change effects. Although there have been studies on Eurasian Gray‐headed Chickadee population declines, inferences from these studies are weakened by distinct aspects of the North American population's range such as the local bird community composition and the limited anthropogenic alterations to habitat (e.g., logging).

Boreal Chickadees are a species of least concern, distributed across northern North America (BirdLife International 2022) and comprised of potentially five subspecies (Ficken et al. 2020). Boreal Chickadees and their sister species the Chestnut‐backed Chickadee ( *P. rufescens* ) form a monophyletic group with the Gray‐headed Chickadee (Harris et al. 2014). The areas of Boreal Chickadee sympatry with the Gray‐headed Chickadee are near the westernmost extent of the Boreal Chickadee distribution, although Boreal Chickadees may have expanded westward in recent decades based upon anecdotal observations (Booms et al. 2020). Studying competition between Boreal Chickadees and Gray‐headed Chickadees is nearly impossible owing to current population densities of the latter species. However, interspecific hybridization can be investigated because it might have left genetic traces in current populations of Boreal Chickadees.

Genetic approaches have been used in several instances to search for introgression into rare bird populations to assess the risk posed by hybridization (e.g., Lawson et al. 2017; Moulton et al. 2017; Yang et al. 2018; Forsdick et al. 2021). Similar methods have become available for use with historical museum samples as DNA sequencing methods appropriate for older samples have been developed (Linck et al. 2017; Raxworthy and Smith 2021). For a few systems in which endangered bird populations in the wild or captivity can be sampled but are of unknown genetic integrity, museum samples have been used to assess past introgression into remnant populations (Baveja et al. 2019, 2021). In the case of an extremely rare, declining bird, historical samples can facilitate the investigation of hybridization and inform conservation decisions even when no contemporary samples of the rare population can be obtained.

We aimed to identify if introgression from Gray‐headed Chickadees into the Boreal Chickadee population has occurred by reduced representation sequencing of sympatric Boreal Chickadees' genomes and comparing those data with sequences from historical samples of both Boreal Chickadees and Gray‐headed Chickadees. Despite the absence of contemporary Gray‐headed Chickadee samples from North America, we leveraged historical samples to infer whether hybridization has occurred in the past and gain insight into aspects such as hybridization frequency as well as hybridization or backcross asymmetry. Variations in the observed patterns of introgression between nuclear DNA (nuDNA) and maternally inherited mitochondrial DNA (mtDNA) can be indicative of specific interbreeding dynamics other than solely identifying the presence or absence of hybrid ancestry. Given the occurrence of hybridization between other *Poecile* species pairs, we hypothesize that there will be evidence of introgression within the contemporary Boreal Chickadee specimens. Additionally, we predict that the observed patterns of introgression will be consistent with a shift from Gray‐headed Chickadees being relatively more abundant than Boreal Chickadees to being relatively less common.

Lastly, we evaluate genetic diversity and structure between species, between time periods, and among sampling sites as well as estimate effective population sizes and model demographic history. Inclusion of descriptive analyses of spatial and temporal genetic diversity enable us to further evaluate the potential influence of introgression on Gray‐headed Chickadees and inform our understanding of the population size changes that occurred for both chickadee species during the period of Gray‐headed Chickadee decline. Ultimately, understanding the spatial and temporal arrangement of genetic diversity can provide insights into whether hybridization played a role in the decline of Gray‐headed Chickadees and also yield reference data on putative pre‐decline conditions that may inform recovery efforts.

## 2 Methods

### 2.1 Sample Collection

**FIGURE 2:**
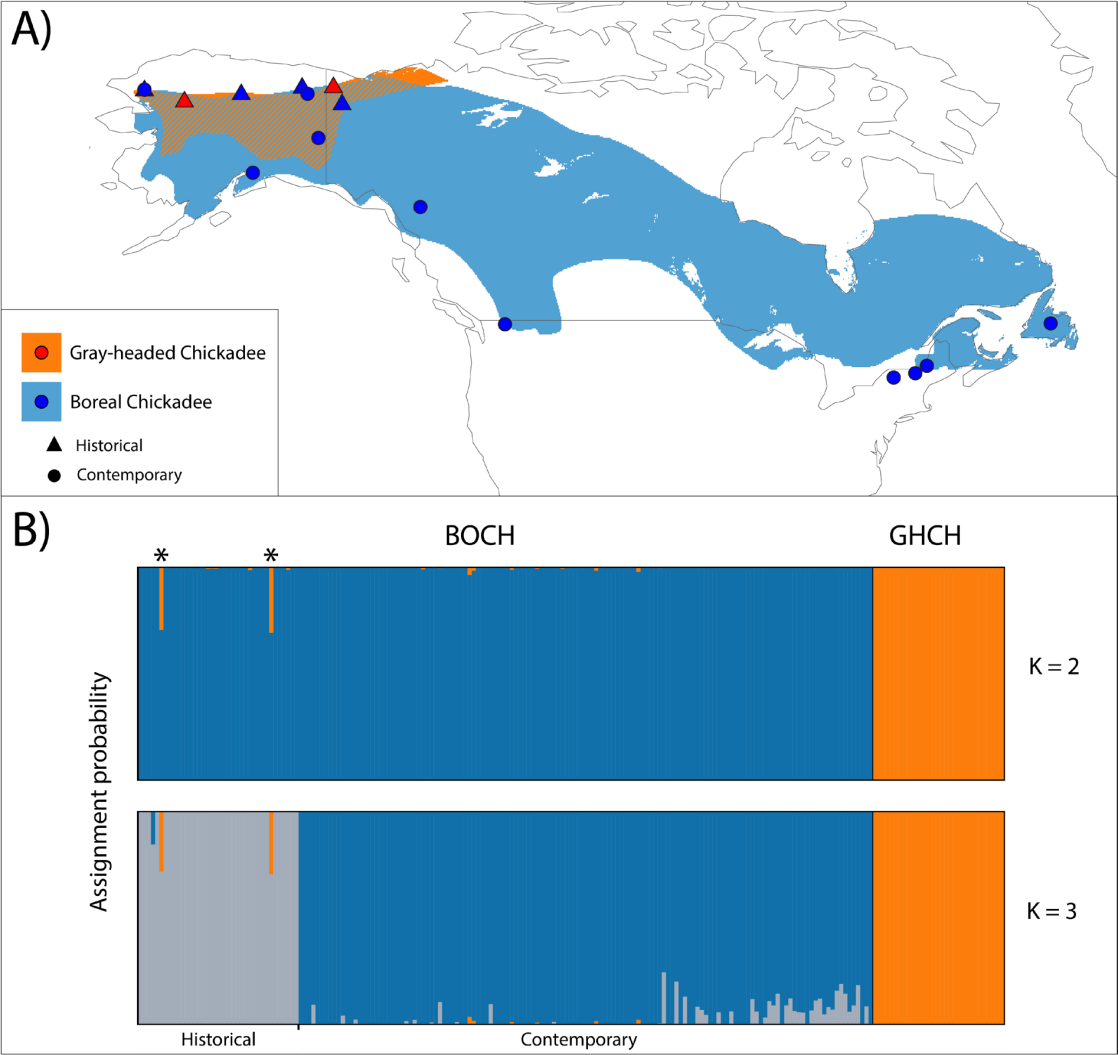
(A) Map of Boreal Chickadee and Gray‐headed Chickadee distributions within North America (“BirdLife International 2022). Historical sampling site locations are represented by triangles and contemporary sampling site locations are represented by circles. Eurasian Gray‐headed Chickadee distribution and sampling location are not shown. (B) Assignment probabilities for *K* = 2 and *K* = 3, the number of clusters resulting in the lowest and second lowest cross‐validation errors, respectively, as estimated in ADMIXTURE. Labels indicate Gray‐headed Chickadees (GHCH), Boreal Chickadees (BOCH), and BOCH sample time period, as well as asterisks to denote the two historical BOCH individuals with assignment probabilities of 0.29 and 0.30 to the orange (GHCH) cluster.

Boreal Chickadee (*n* = 175) and Gray‐headed Chickadee (*n* = 31) samples were obtained from historical (1875–1979) and contemporary time periods (1990–2023) through a combination of trapping efforts and museum tissue loans across 14 sites in North America and Russia (Figure 2A and Table A1 in Appendix A). Historical Gray‐headed Chickadees were obtained from one Alaska site and one Yukon site, while historical Boreal Chickadees were obtained from four Alaska sites and one Yukon site in areas of sympatry with the Gray‐headed Chickadee. It is not possible to obtain contemporary Gray‐headed Chickadee samples from North America as they are extremely difficult to find today, however contemporary eastern Russia Gray‐headed Chickadee samples of the subspecies *P. c. cinctus* were included in analyses. Contemporary Boreal Chickadees from three sites that are sympatric with the historical range of the Gray‐headed Chickadee were sampled. In addition, contemporary Boreal Chickadees were sampled from seven allopatric sites, three of these sites in western North America and four in eastern North America. Blood samples were collected at the contemporary Alaska sites by trapping efforts (U.S. Geological Survey Bird Banding Lab Permit: 24111) and muscle (contemporary) or toe‐pad (historical) tissues from all other sites were obtained through museum loans.

### 2.2 DNA Extraction

DNA was extracted from the contemporary samples using a Qiagen DNeasy blood and tissue extraction kit. Historical sample DNA extraction was performed using a phenol–chloroform protocol (Green and Sambrook 2017) with a Microcon centrifugal filter (Millipore, Burlington, MA) for DNA recovery. Extraction blanks were included for the phenol–chloroform protocol (one extraction blank per 11 DNA extractions). DNA concentration was quantified with a Qubit fluorometer. Processing of historical samples was completed in a dedicated pre‐PCR laboratory. Extraction blanks and subsequent library preparations did not yield any measurable DNA (< 1 ng/μL) and were not included in Illumina sequencing.

### 2.3 Mitochondrial DNA Preparation and Sequencing

Amplification of the mtDNA control region was performed using primers LmochCR1/H1015chCR (Lait and Burg 2013). The control region for historical samples was amplified in five smaller overlapping segments to compensate for the degradation of DNA from older samples (Table A2 in Appendix A). Two of the amplification primers used for these segments were replaced with different primers for sequencing to avoid a section of repeated nucleotides near the start of sequences. Successful amplification was assessed using gel electrophoresis; PCR products were sequenced on an ABI 3730xl instrument (Functional Biosciences Sequencing, Madison, WI), and forward and reverse sequences were reconciled in Sequencher (Gene Codes Corporation, Ann Arbor, MI).

### 2.4 DNA Library Preparation and Sequencing

Contemporary samples were processed using a double digest restriction site‐associated DNA (ddRAD) sequencing approach based on a protocol by DaCosta and Sorenson (2014). DNA was digested with restriction enzymes Sbf1 and EcoR1, sequencing adapters and barcodes were ligated to the fragments, and a double‐sided size selection protocol using SPRI beads (Beckman Coulter Inc., Brea, CA) was performed to select for adapter ligated DNA fragments between 300 and 450 base pairs (bp) (Sonsthagen et al. 2019). Size‐selected libraries were amplified using PCR and purified with AMPure XP beads. PCR product concentrations were quantified with fluorometry and diluted before being combined in a final library which was sequenced on an Illumina HiSeq 4000 with single‐end 150 bp reads.

Data from historical samples were collected using a hybridization capture protocol to sequence restriction‐site associated DNA (hyRAD) loci. HyRAD targets ddRAD loci using bait capture probes that are short in length, allowing for hybridization to sample DNA before sequencing to avoid issues when DNA has degraded due to age and does not span an entire ddRAD locus (Suchan et al. 2016). Sequence data collected from Anchorage Boreal Chickadee samples were used to design capture baits. ddRAD libraries were generated as described above with read processing and ddRAD locus identification following DaCosta and Sorenson (2014). Briefly, reads were filtered by quality, clustered into putative loci with USEARCH (Edgar 2010), and within locus alignment performed with MUSCLE (Edgar 2004). Candidate baits were designed by Arbor Biosciences (Daicel Arbor Biosciences, Ann Arbor, MI). Bait sequences were 70 bp in length with 4× tiling for each ddRAD locus. Bait sequences that mapped to a reference Black‐capped Chickadee genome (GenBank assembly accession GCA_011421415.1) were synthesized as biotinylated probes for hybridization capture with the degraded historical samples.

Libraries from historical samples were prepared using a Claret Bioscience PicoPlus library preparation kit following the ancient DNA protocol (Claret Bioscience, Santa Cruz, CA) with two modifications: Kapa HiFi HotStart Uracil+ DNA Polymerase (Kapa Biosystems, Wilmington, MA) and associated manufacturer recommended thermocycler profile replaced the PCR master mix provided in the kit and thermocycler conditions for the PCR index step. PCR amplification was performed for 13 cycles. Libraries were quantified using a Kapa Biosystems library quantification kit. Libraries with similar concentrations were paired and pooled in equimolar amounts for hybridization capture. The MyBaits capture protocol (Daicel Arbor Biosciences) was followed. Briefly, two rounds of sample hybridization at 58°C (for 22.5 and 17 h, respectively) were performed to increase target read capture. Capture pools were quantified (as above), pooled in equimolar concentrations, and sequenced on an Illumina HiSeq 4000 with 100 bp paired‐end reads.

### 2.5 Read Assembly

Sequence reads from all historical and contemporary samples were processed using a custom python script (Lavretsky et al. 2020), with modifications for the use of paired‐end reads and single‐end reads in a single analysis. Historical paired‐end reads were merged using PEAR (Zhang et al. 2014) prior to the trimming and filtering of reads with Trimmomatic (Bolger et al. 2014). The bwa‐mem algorithm was used to align reads to the reference loci which were used to construct hyRAD capture baits (Li 2013), after which the reads were combined with the Samtools mpileup function (Li et al. 2009). MapDamage 2.0 was used to check historical samples for signs of post‐mortem sequence degradation and rescale quality scores for samples with evidence of damage (Jónsson et al. 2013).

The output Variant Call Format (VCF) file was filtered using vcftools and the following filtering parameters to produce a dataset with full read data for variable and nonvariable sites: a minimum genotype depth of 10 reads, a max missingness of 20% per site, and an allelic depth of 4 per site (Danecek et al. 2011). Different filter parameters were applied to the full sequence data to generate four single nucleotide polymorphism (SNP) datasets for subsequent analyses: *Chickadee_SNPs*, *Chickadee_SNPs_rare*, *Chickadee_randomSNPs*, and *Chickadee_randomSNPs_rare*. The full sequence data were filtered to only bi‐allelic SNPs with a minor allele frequency (maf) greater than 0.0025, an allelic depth of 4, a minimum QUAL score of 30, and a max missingness of 10% [*Chickadee_SNPs*]. The maf filter removes allelic variants with only a single copy across samples to lower potential sequencing error effects, but a second SNP dataset was created with the same filtering parameters excluding any maf threshold [*Chickadee_SNPs_rare*], for use with analyses that are informed by rare variants. A perl script was used to make a version of each of these two SNP datasets that contains one randomly selected SNP per locus [*Chickadee_randomSNPs*, *Chickadee_randomSNPs_rare*], for use with analyses that assume no linkage of alleles (https://github.com/santiagosnchez/sing_snp_vcf).

### 2.6 Genetic Diversity and Structure

Haplotype and nucleotide diversities, and neutrality statistics from mtDNA for each sampling site were calculated using Arlequin v3.5 (Excoffier and Lischer 2010). Expected and observed heterozygosities were also calculated for each sampling site from the ddRAD [*Chickadee_SNPs*] dataset using the *snpR* R package in program R version 4.3.2 (Hemstrom and Jones 2023; R Core Team 2023). Additionally, we constructed an mtDNA haplotype network using median‐joining in Network v10.2 to visualize genetic diversity within and among Boreal Chickadees and Gray‐headed Chickadees (Bandelt et al. 1999). To quantify genetic differentiation between sampling sites, we calculated pairwise *Φ*ST values using mtDNA haplotypes in Arlequin and pairwise *F*_ST_ values using the [*Chickadee_SNPs*] dataset in *snpR*.

We further assessed genetic structure at mtDNA by grouping the sampling sites into five broader groups: the Gray‐headed Chickadees, the historical Boreal Chickadees, the contemporary sympatric Boreal Chickadees, the contemporary allopatric western Boreal Chickadees, and the contemporary allopatric eastern Boreal Chickadees. A hierarchical analysis of molecular variance (AMOVA) was performed in Arlequin to inform the relative amounts of mtDNA differentiation between and within these groupings to help identify biological populations represented by our sampling sites. Three additional AMOVAs were run with subsets of those groups to evaluate relationships of interest between several temporal and geographic divisions, namely whether there is differentiation between historical and contemporary sympatric Boreal Chickadees, between contemporary western Boreal Chickadees in areas of sympatry and allopatry, and between contemporary allopatric Boreal Chickadees on the western and eastern sides of the continent.

Genetic structure was investigated using two clustering approaches with our ddRAD datasets. Clustering of individuals based on maximum likelihood assignments was performed in ADMIXTURE within the AdmixPipe V3 pipeline on [*Chickadee_randomSNPs*] (Alexander and Lange 2011; Mussmann et al. 2023). Ten replicates of *K* 1–10 clusters were performed, and the best fitting number of clusters was assessed by minimizing the CV (cross‐validation) score. Individuals with admixture assignments less than 100% at *K* = 2 were identified as putative mixed ancestry for further analyses. Principal Component Analyses (PCAs) were run in *snpR* on [*Chickadee_SNPs*] based on all chickadee samples, and PCA results were visualized by plotting the first two principal components.

### 2.7 Hybrid Classification

We utilized two approaches to characterize individuals with putative mixed ancestry. First, we simulated crosses between putative pure individuals to classify test samples as being from the parental class, an F1 hybrid, and F2 hybrid, or a first‐generation backcross into either parental population using *hybriddetective*, an R package based on the program NewHybrids (Anderson and Thompson 2002; Wringe et al. 2017). We divided [*Chickadee_SNPs*] into putative pure and putative mixed sample subsets and converted both VCF files to genepop format using PGDSpider V.2.1.1.5 (Lischer and Excoffier 2012). Using an Rscript by Zbinden et al. (2023) to run *hybriddetective* analyses, we first selected a diagnostic panel of 200 SNPs out of linkage disequilibrium (*r* ^2^ < 0.2) that are differentiated (*F*_ST_ > 0.05) between the putative pure Boreal Chickadee and putative pure Gray‐headed Chickadee samples, and then simulated 10 individuals of each hybrid class using random allele sampling. We estimated the posterior probability of assignment to each hybrid class for the putative mixed individuals by Monte Carlo Markov chain simulation with a burn‐in of 250,000 followed by 1,000,000 sweeps (Anderson and Thompson 2002).

To assist with characterizing potential introgression in the putative mixed ancestry individuals which may not be recent‐generation hybrids, we used the *gghybrid* R package. This Bayesian method estimates a hybrid index for all samples which represents the proportion of a genome sourced from one of the predefined parental populations (Bailey 2024). We converted [*Chickadee_randomSNPs*] to STRUCTURE format using PGDSpider, and labeled individuals as Boreal Chickadees, Gray‐headed Chickadees, or putative mixed individuals. SNPs were filtered using the AF.CIoverlap = FALSE filter to remove sites with similar allele frequency between Boreal Chickadees and Gray‐headed Chickadees, and a burn‐in of 2000 was followed by 3000 additional iterations. Hybrid index scores were visualized with 95% credible intervals for each sample, as well as a credible inner limit for each parent population which was the innermost 95% credible interval for any reference pure individual. Two additional replicates with the same settings were also performed to assess convergence with the Gelman‐Rubin convergence diagnostic (Gelman and Rubin 1992).

### 2.8 Gene Flow

Gene flow assessment was performed on nuDNA using fineRADstructure (Malinsky et al. 2018) and dadi (Gutenkunst et al. 2009). FineRADstructure creates co‐ancestry matrices between individuals based on coalescence theory and nearest neighbor relationships at each locus, providing a pairwise co‐ancestry coefficient that can indicate relatively recent gene flow. We used the initial filtered dataset including nonvariable sites as the input file for fineRADstructure by conversion with the RADpainter hapsFromVCF command with default parameters. The sampleLD.R script was also used to reorder loci according to linkage disequilibrium values, as recommended for unmapped loci by Malinsky et al. (2018). Co‐ancestry plots were produced with the fineSTRUCTURE GUI program (https://people.maths.bris.ac.uk/~madjl/finestructure/finestructure.html).

Dadi, which uses a diffusion approximation approach to infer demographic history from site frequency spectra, was used to model and compare fits between 10 proposed evolutionary models of two‐population gene flow (Gutenkunst et al. 2009). We compared results from models run on the historical Gray‐headed Chickadee and historical Boreal Chickadee samples (hereafter Set A) with models run on the historical Gray‐headed Chickadee and contemporary sympatric Boreal Chickadee samples (hereafter Set B). Tests were performed with the dadi_pipeline tool, using folded site frequency spectra produced from [*Chickadee_randomSNPs_rare*] after input datasets were subset to the desired individuals (Portik et al. 2017). Recent‐generation hybrids (F1, F2, or first‐generation backcross) identified by *hybriddetective* were manually removed. Optimizations were run with a grid size of [80, 90, 100] and down‐projection values for each population set that maximized the number of segregating sites (i.e., [58, 36] for Set A and [120, 34] for Set B). Log‐likelihood values were compared between the proposed models which included differing demographic dynamics such as no migration, symmetric migration, asymmetric migration, secondary contact, and an instantaneous population size change. Optimized parameter values for the best‐fitting models were converted to biologically meaningful values using an estimated generation time of 2.26 years (Willow Tit ( *P. montanus* ); Kvist et al. 1998) and nuclear mutation rate of 2.3 × 10^−9^ mutations/site/year (Collared Flycatcher ( *Ficedula albicollis* ); Smeds et al. 2016).

### 2.9 Effective Population Size Estimates

Estimates of effective population size (*N*_e_) and associated jackknife confidence intervals were performed using the linkage disequilibrium method in NeEstimator v2.1 (Do et al. 2014) to assess genetic support for the prior observation that Boreal Chickadee abundance in areas of sympatry may have increased in recent decades (Booms et al. 2020). This analysis was carried out on [*Chickadee_randomSNPs_rare*] with the *snpR* calc_ne function. A series of four maf thresholds were selected (*P*_crit_ = 0, 0.03, 0.06, 0.09) that lead to functional changes in filtered loci. Lower *P*_crit_ values lead to greater precision but also an unconservative estimate of *N*_e_, so a series of different *P*_crit_ values was used to assess the consistency of *N*_e_ estimates (Waples and Do 2008). Further, variance in *N*_e_ estimates across a range of *P*_crit_ values is suggestive of a history of gene flow and (or) presence of first‐generation dispersers, whereas stable *N*_e_ estimates across a range of *P*_crit_ values are suggestive of isolated populations (Waples and England 2011). Contemporary Boreal Chickadee samples from areas of sympatry with the Gray‐headed Chickadees and the western allopatric samples were combined for the contemporary western Boreal Chickadee *N*_e_ estimate. Results were similar when only sympatric Boreal Chickadee sampling sites were included.

## 3 Results

### 3.1 Read Assembly

Capture baits were generated from 6436 reference loci. After the initial read quality filtration step but prior to retention of only variable sites, 822,806 bp across 6000 loci remained with a median read depth per individual sample of 114.4 (35.5–192.9). The median read depth per individual was 146.6 for historical samples and 111.1 for contemporary samples. After filtering to include only bi‐allelic SNPs without any maf filter, 54,839 SNPs from 5373 loci remained [*Chickadee_SNPs_rare*]. When SNPs were also filtered based on a minimum maf of 0.0025, 40,580 SNPs from 5363 loci were retained [*Chickadee_SNPs*].

### 3.2 Genetic Diversity and Divergence

Nucleotide diversity at mtDNA control region ranged from 0.0005 to 0.0043 (Table 1). Tajima's *D* values were non‐significant except for Boreal Chickadee samples at three locations with significantly negative values: historical Old Crow River, Alaska; contemporary Kelly River, Alaska; and contemporary Newfoundland, Canada. Observed heterozygosities for ddRAD SNPs ranged from 0.0419 to 0.0807 and were higher for Boreal Chickadee sampling sites than for Gray‐headed Chickadee sampling sites. Observed heterozygosities were also higher for historical samples than contemporary samples within each species, both at the resampled Kelly River and Sheenjek locations as well as all other sampling site comparisons (Table 1).

**TABLE 1:** Sample location, sizes, and genetic diversity metrics for Boreal Chickadee (BOCH) and Gray‐headed Chickadee (GHCH) from mitochondrial DNA (mtDNA) control region and nuclear DNA (nuDNA) biallelic single‐nucleotide polymorphisms (*n* = 40,580) identified from double digest restriction‐site associated DNA loci, including number of haplotypes, nucleotide diversity, Tajima's *D*, observed heterozygosity (*H*_O_), and expected heterozygosity (*H*_E_).

Group	Species	Sampling site	Time period	mtDNA				nuDNA		
				*n*	Haplotype number	Nucleotide diversity	Tajima's *D*	*n*	*H*_O_	*H*_E_
1	BOCH	Kobuk River, AK, USA	Historical	13	5	0.0010	−1.4	13	0.0751	0.0713
	BOCH	Kelly River, AK, USA	Historical	3	3	0.0026	0.0	3	0.0807	0.0682
	BOCH	Interior Alaska, USA	Historical	6	2	0.0008	0.0	6	0.0738	0.0640
	BOCH	Old Crow River, Yukon, Canada	Historical	9	6	0.0014	**−1.7**	9	0.0751	0.0700
	BOCH	Sheenjek and Coleen, AK, USA	Historical	7	2	0.0007	1.3	7	0.0718	0.0661
2	BOCH	Kelly River, AK, USA	Contemporary	59	11	0.0012	**−1.5**	59	0.0691	0.0711
	BOCH	Sheenjek and Coleen, AK, USA	Contemporary	5	4	0.0031	−1.1	5	0.0686	0.0633
	BOCH	Chicken, AK, USA	Contemporary	13	3	0.0009	0.1	13	0.0687	0.0682
3	BOCH	Anchorage, AK, USA	Contemporary	25	6	0.0013	−1.1	24	0.0693	0.0702
	BOCH	British Columbia, Canada	Contemporary	3	2	0.0009	0.0	3	0.0689	0.0596
	BOCH	Washington, USA	Contemporary	5	2	0.0005	−0.8	5	0.0646	0.0622
4	BOCH	Newfoundland, Canada	Contemporary	6	6	0.0042	**−1.4**	6	0.0637	0.0607
	BOCH	Maine, USA	Contemporary	10	7	0.0027	−1.4	10	0.0682	0.0661
	BOCH	New York, USA	Contemporary	8	6	0.0042	−1.3	8	0.0670	0.0647
	BOCH	Vermont, USA	Contemporary	3	2	0.0043	0.0	3	0.0692	0.0483
5	GHCH	Kobuk River, AK, USA	Historical	10	4	0.0014	−0.9	11	0.0494	0.0477
	GHCH	Old Crow River, Yukon, Canada	Historical	12	4	0.0016	−0.9	12	0.0498	0.0450
	GHCH	Russia	Contemporary	8	6	0.0043	−0.7	8	0.0419	0.0389

*Note:* The time period for each sampling site is listed: Historical (1875–1979) and contemporary (1990–2023). Group numbers denote populations that were grouped to test hypotheses regarding partitions of the genetic structure. Significant Tajima's *D* values (*p* < 0.05) are in bold text.

**FIGURE 3:**
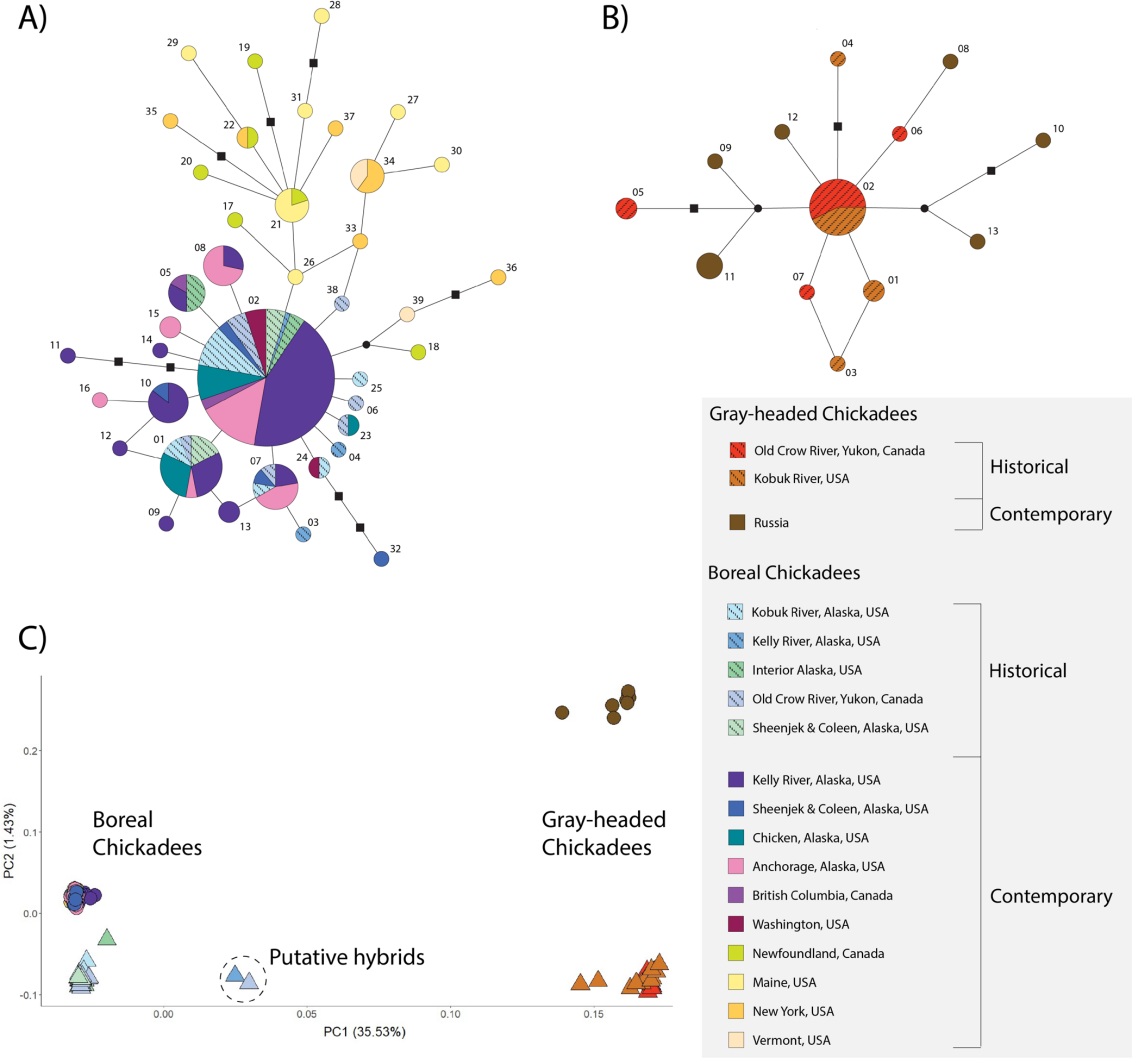
Haplotype network of mitochondrial DNA (mtDNA) control region for (A) Boreal Chickadees and (B) Gray‐headed Chickadees. Haplotype nodes are scaled with occurrence in the dataset, black nodes depict unsampled haplotypes, hashed lines indicate historical samples, and labels indicate haplotype. (C) PCA using nuclear DNA (nuDNA) bi‐allelic single nucleotide polymorphisms generated from double digest restriction‐site associated DNA sequence loci. Triangle symbols represent historical samples and circle symbols represent contemporary samples. Two putative hybrids located intermediate between the two species‐based clusters along PC1 are labeled.

Gray‐headed Chickadee and Boreal Chickadee mtDNA control region haplotypes were separated by 27 variant sites (Figure 3). There were no shared haplotypes between western and eastern Boreal Chickadee samples (Figure 3A). Within Boreal Chickadees from Alaska and northwestern Canada, there were five private haplotypes in historical samples and 10 private haplotypes in contemporary samples. There were shared haplotypes between the two historical Gray‐headed Chickadee sampling sites but not with the contemporary Russia Gray‐headed Chickadee samples (Figure 3B).

Grouping of sampling sites into Gray‐headed Chickadee sites, historical Boreal Chickadee sites, contemporary sympatric Boreal Chickadee sites, contemporary allopatric western Boreal Chickadee sites, and contemporary eastern Boreal Chickadee sites explains a significant portion of the genetic structure, with an among‐group differentiation value (*Φ*_CT_) of 0.874 (Table A3 in Appendix A). A *Φ*_CT_ value of 0.461 was estimated between contemporary allopatric western and eastern Boreal Chickadees, but *Φ*_CT_ was not significant between sympatric Boreal Chickadees in the historical and contemporary datasets or between the sympatric and allopatric contemporary western Boreal Chickadees.

**FIGURE A1:**
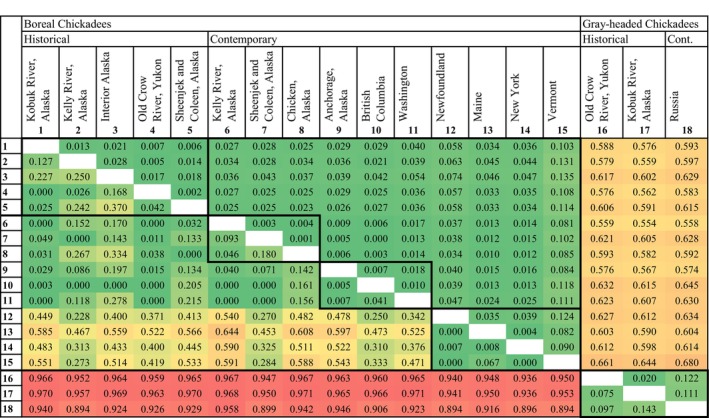
Estimates of pairwise differentiation across sampling sites based on mitochondrial DNA control region sequence data (*Φ*_ST_; below diagonal) and biallelic single nucleotide polymorphisms generated from double digest restriction site associated DNA data (*F*_ST_; above diagonal). Sampling sites are grouped based on time period: Historical (1875–1979) and contemporary (1990–2023). Boxes outline comparisons within historical Boreal Chickadees, within contemporary sympatric Boreal Chickadees, within contemporary allopatric western Boreal Chickadees, within contemporary eastern Boreal Chickadees, and within Gray‐headed Chickadees. Negative estimates are displayed as zeroes, and colors are on a green to red gradient from 0 to 1.

Differentiation among the Gray‐headed Chickadee and Boreal Chickadee sites was observed at both the mtDNA *Φ*_ST_ and nuDNA *F*_ST_ estimates (Figure A1 in Appendix A). MtDNA Φ_ST_ values are generally higher than the corresponding nuDNA *F*_ST_ values. Consistent with AMOVA analyses, both mtDNA and nuDNA estimates of differentiation are higher between eastern Boreal Chickadee sites and western Boreal Chickadee sites than within western and within eastern Boreal Chickadees or between historical and contemporary time periods within Alaska and northwestern Canada.

### 3.3 Genetic Structure

Cross‐validation scores were minimized at *K* = 2 in our ADMIXTURE analysis. Boreal Chickadee individuals and Gray‐headed Chickadee individuals formed species‐specific clusters (Figure 2B). The second most supported number of clusters was 3, with substructure observed within Boreal Chickadee as individuals clustered based on time period. At *K* = 2, two historical Boreal Chickadees had approximately 30% ancestry fraction from the Gray‐headed Chickadee cluster: BOCH‐158359 from Kelly River, Alaska, and BOCH‐468112 from Old Crow River, Yukon. Of the 15 additional Boreal Chickadee samples with marginal (all < 5%) non‐zero assignment probabilities to the Gray‐headed Chickadee cluster, five were historical samples and 10 were contemporary samples. The five historical samples included three individuals from Kobuk River, Alaska, one individual from Interior Alaska, and one individual from Old Crow River, Yukon. The 10 contemporary samples included nine individuals from Kelly River, Alaska, and one individual from Chicken, Alaska. All 17 individuals were marked as having putative mixed ancestry for subsequent analyses. The other 188 individuals with 100% assignment to a cluster in ADMIXTURE were used as reference putative pure individuals in subsequent analyses (described below).

Visualization of the PCAs is concordant with the patterns observed with the ADMIXTURE plots. The first principal component (PC1) accounted for 35.53% of variation and individuals clustered by species, except for the two individuals (BOCH‐158359 and BOCH‐468112) which had intermediate placement between the species clusters (Figure 3C). PC2 accounted for 1.43% of variation and chickadees clustered loosely by temporal sampling period, although the greatest separation along the y‐axis is between historical and contemporary Gray‐headed Chickadees that are also spatially isolated. Within Gray‐headed Chickadees, individuals clustered by subspecies. Within Boreal Chickadees, individuals clustered primarily by time period.

### 3.4 Hybrid Classification

**FIGURE 4:**
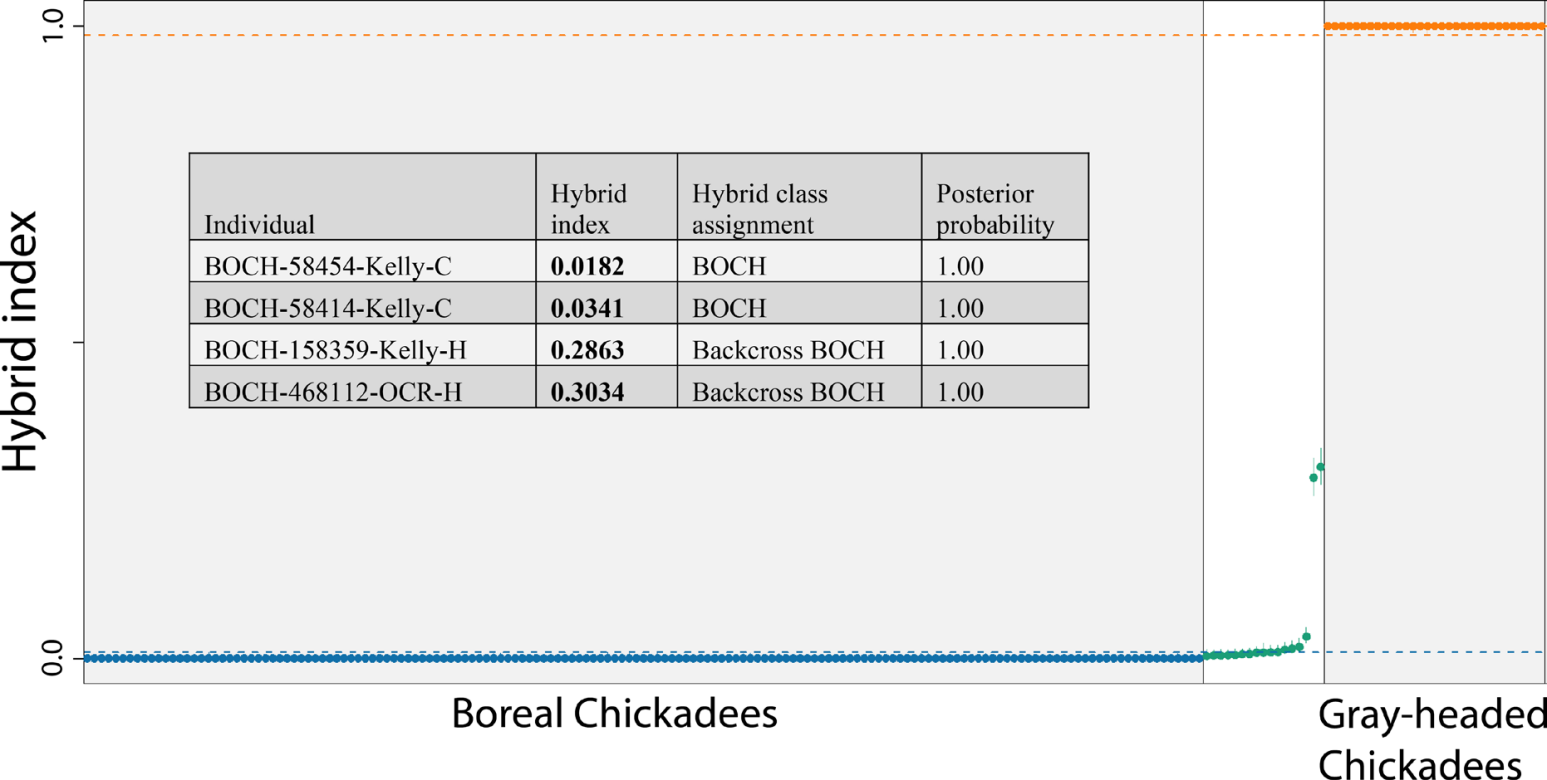
Plot of hybrid index scores for all individuals using *gghybrid*. Individuals with no mixed assignment in the ADMIXTURE analysis at *K* = 2 were treated as reference pure populations for their respective species. Error bars indicate 95% posterior credible intervals for hybrid index scores. Horizontal lines depict inner limit credible thresholds for pure parental populations, resulting from picking the innermost 95% posterior credible interval for any sample in the two parent populations. Hybrid class assignment probabilities are listed for Boreal Chickadees (BOCH) with significant hybrid index values.

The hybrid class assignment analysis using *hybriddetective* classified both BOCH‐158359 and BOCH‐468112 individuals to the Boreal Chickadee backcross class, and the 15 individuals with marginal assignment probabilities to the pure Boreal Chickadee class (Figure 4 and Table A4 in Appendix A). We then used *gghybrid* to estimate a hybrid index score relative to the parental populations. The *gghybrid* simulations consistently converged according to the convergence diagnostic test, as only two samples had a Gelman‐Rubin score over 1.2. Four Boreal Chickadee samples had significant hybrid indices, two being the putative Boreal Chickadee backcrosses BOCH‐158359 and BOCH‐468112 as well as the two contemporary Boreal Chickadees BOCH‐58454 and BOCH‐58414 from Kelly River, Alaska (Figures 1 and 4). These four individuals had a 95% credible interval which did not overlap with the highest credible interval upper bound of any putative pure Boreal Chickadee.

### 3.5 Gene Flow

**FIGURE 5:**
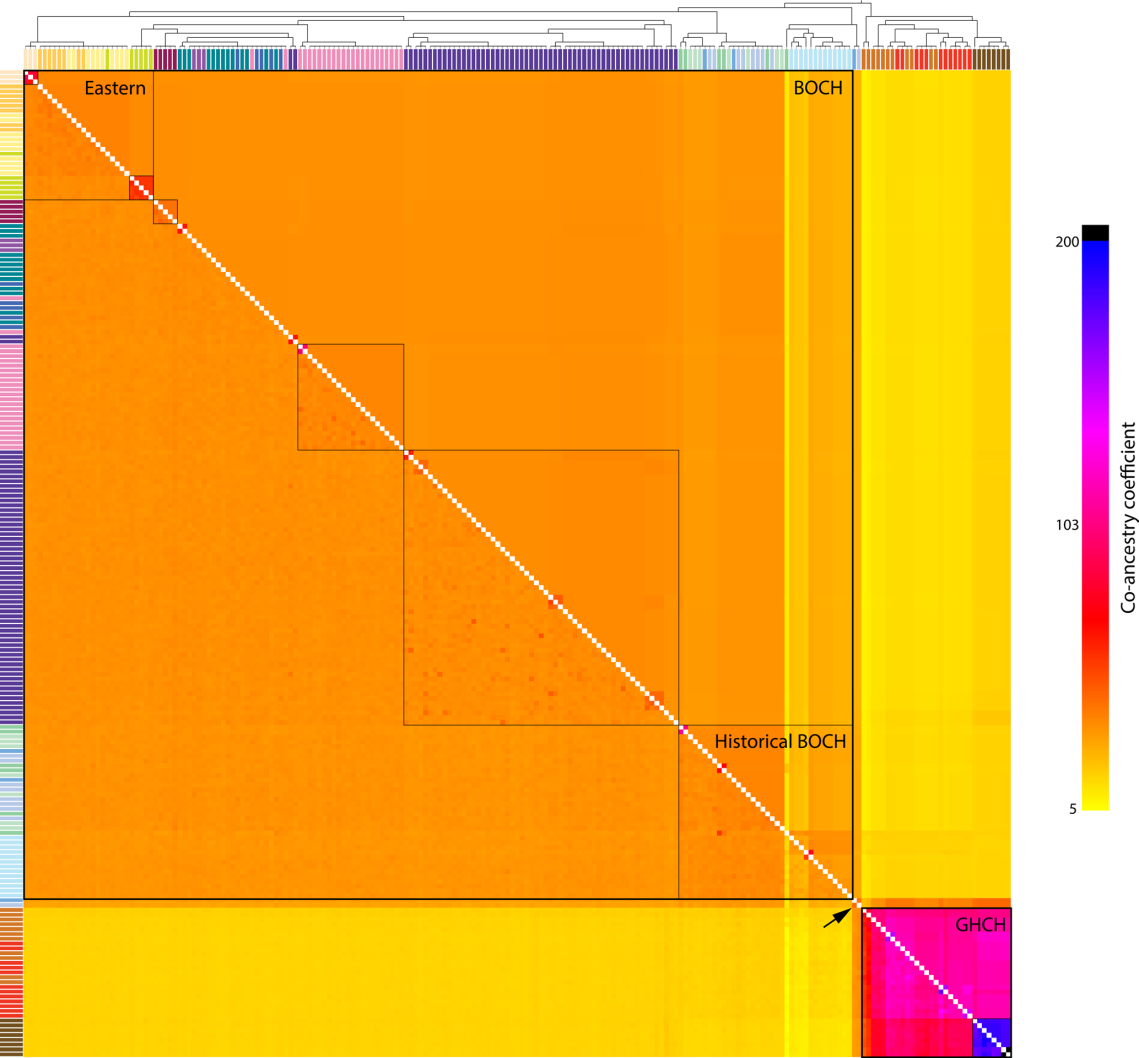
Co‐ancestry matrix generated from double digest restriction‐site associated DNA (ddRAD) sequence loci in fineRADstructure. Co‐ancestry values for individual pairwise comparisons are displayed below the diagonal whereas co‐ancestry value means for each dendrogram node are displayed above the diagonal. Black squares denote genetic groupings within Boreal Chickadees (BOCH) and Gray‐headed Chickadees (GHCH), and the arrow points at the two putative hybrid historical BOCH. Sampling site color labels follow Figure 3.

The observed co‐ancestry coefficient patterns were consistent with the findings of the population structure and hybridization analyses. Boreal Chickadees and Gray‐headed Chickadees had greater similarity to other individuals assigned to the same species, except for the two putative backcross Boreal Chickadees BOCH‐158359 and BOCH‐468112, which had relatively consistent pairwise co‐ancestry coefficients across all samples irrespective of species (Figure 5). High similarity was estimated for six groups, which coincide with regional or temporal groups: eastern contemporary Boreal Chickadees, historical Boreal Chickadees, contemporary Boreal Chickadees from Kelly River, Alaska, contemporary Boreal Chickadees from Anchorage, Alaska, contemporary Boreal Chickadees from Washington, and the Gray‐headed Chickadees. Within those groups, there is high similarity among the Russia Gray‐headed Chickadees, among the Vermont Boreal Chickadees, and among the Newfoundland Boreal Chickadees.

**FIGURE 6:**
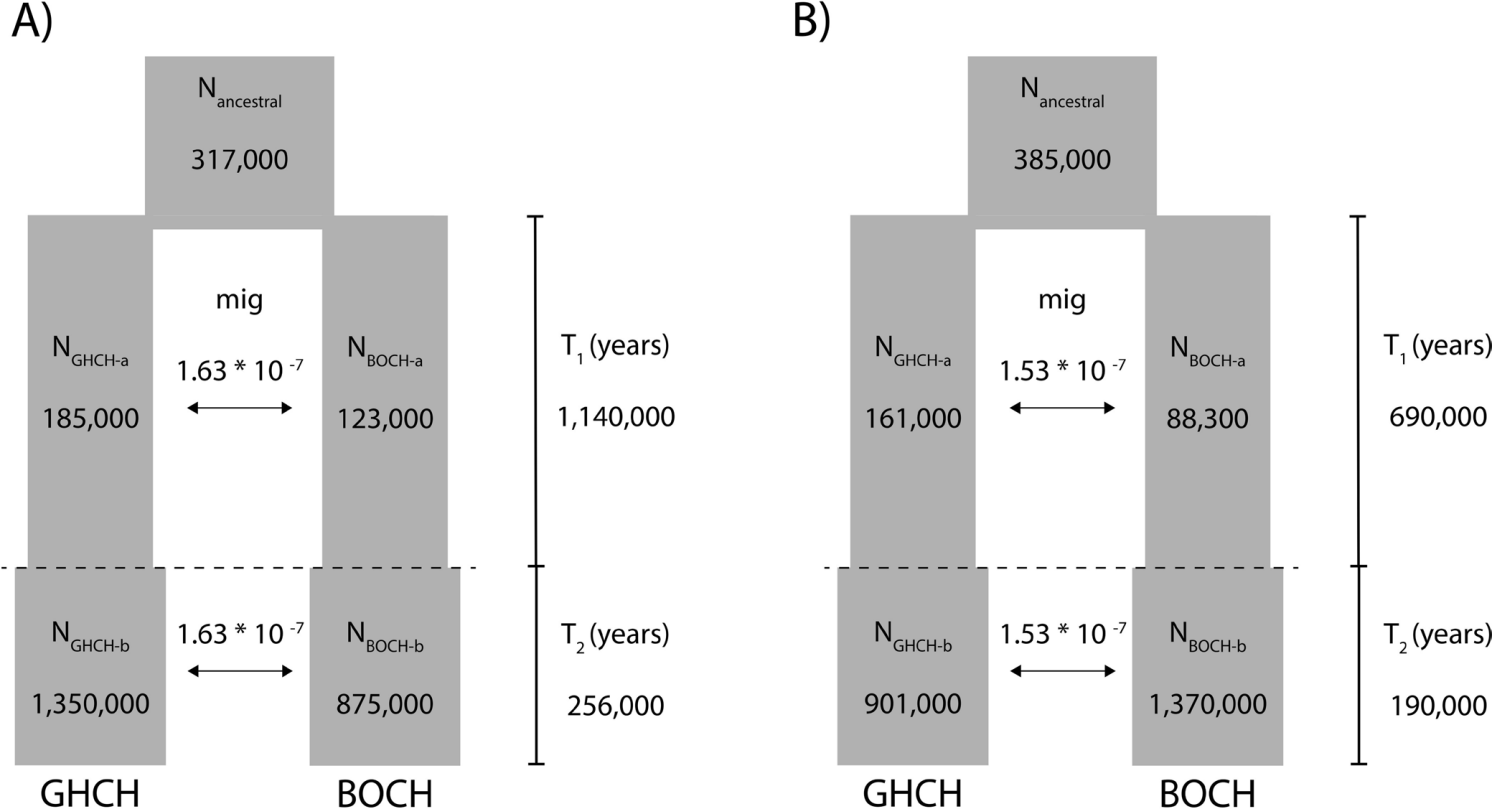
Biological parameters derived from the most likely dadi model for (A) the historical Gray‐headed Chickadee (GHCH)—historical Boreal Chickadee (BOCH) dataset and (B) the historical GHCH—contemporary sympatric BOCH dataset. Both models represent two‐population divergence with continuous symmetrical migration and an instantaneous size change [sym_mig_size]. *T*_1_ is the time estimate in years from divergence until the instantaneous size change, *T*_2_ is the time estimate in years from instantaneous size change until present, mig is the estimated proportion of each population that are new migrants each generation, and *N* values are population size estimates. Figure layout is taken from dadi_pipeline documentation with modifications (Portik et al. 2017). Dimensions are not scaled with values.

**FIGURE A2:**
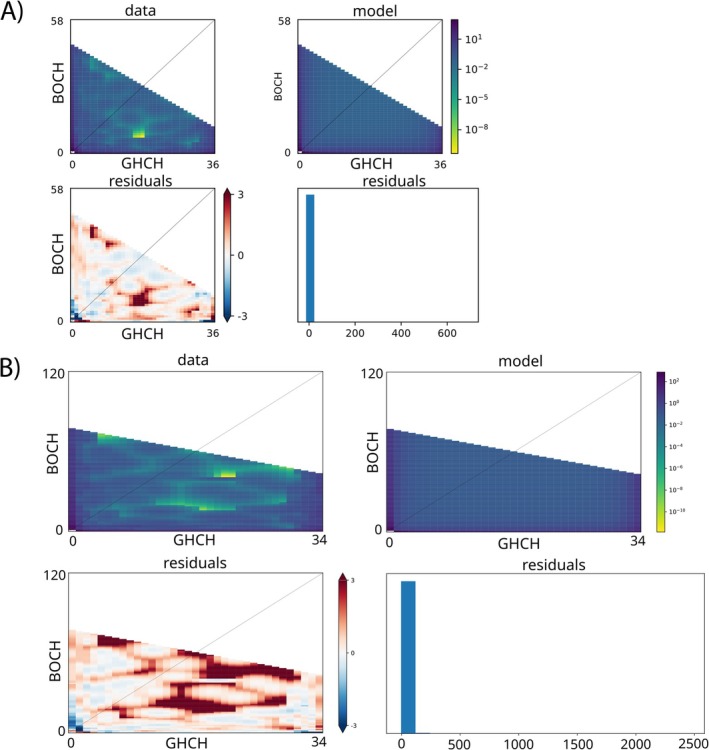
Dadi diagnostic plots for the highest likelihood model for (A) the historical Gray‐headed Chickadee (GHCH)—historical Boreal Chickadee (BOCH) dataset and (B) the historical GHCH—contemporary sympatric BOCH dataset was symmetrical migration with an instantaneous size change. In (A) and (B), the top left plot shows the observed site frequency spectra, the top right plot shows the modeled site frequency spectra, the bottom left shows the residual spectra, and the bottom right shows the distribution of residual magnitude.

The most likely two‐population demographic model using either historical Boreal Chickadees in Set A or contemporary sympatric Boreal Chickadees in Set B as dadi input data was symmetrical migration with an instantaneous size change (Table A5 in Appendix A). The full order of model ranking varied between the two datasets, but models without migration consistently had relatively low likelihood compared to models that included migration. Inspection of residuals for both of the best fitting models showed normally distributed residuals barring a few outliers and a moderately random pattern of dispersal across the site frequency spectrum (Figure A2 in Appendix A). Conversion of optimized parameters for these top models yielded divergence estimates of approximately 1 thousand years ago (kya) for Set A and 880 kya for Set B (Figure 6A,B). The two models indicate a large instantaneous population increase 190 kya or 256 kya for both species. A similar symmetrical migration rate was identified for both Set A and Set B.

### 3.6 Effective Population Size Estimates

**FIGURE 7:**
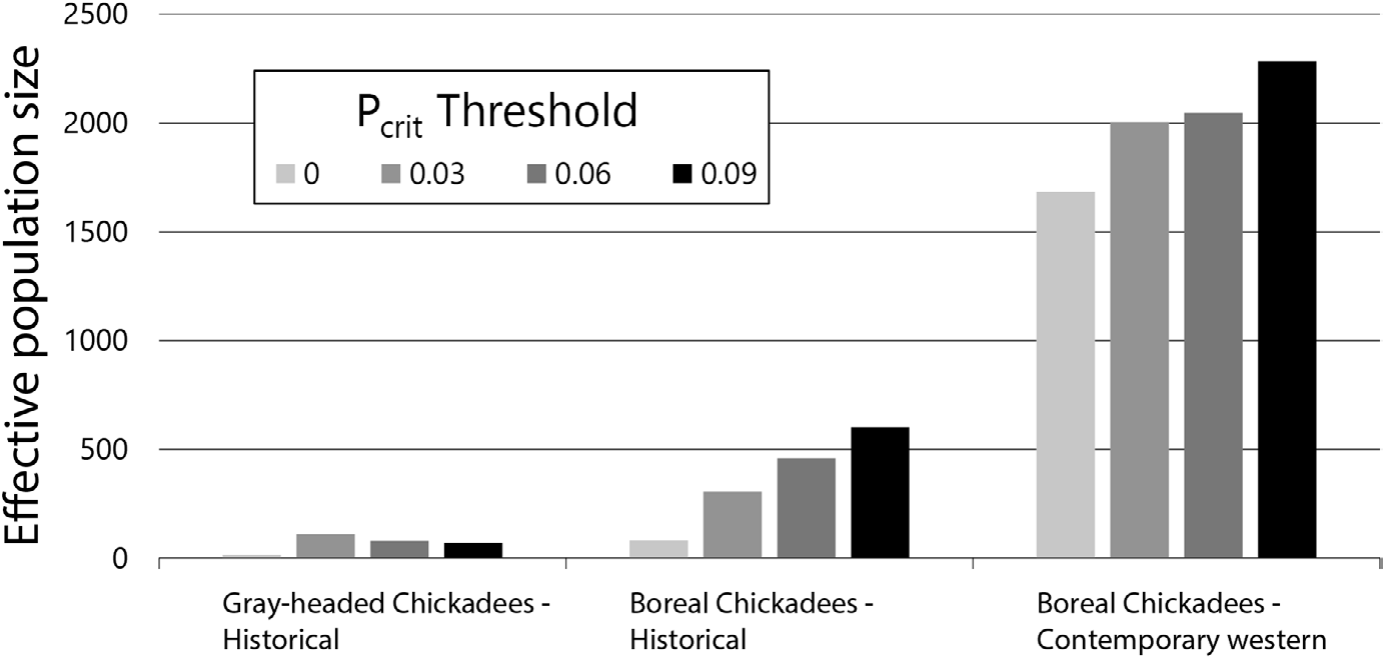
Effective population size estimates for historical Gray‐headed Chickadee samples, historical Boreal Chickadee samples, and contemporary western Boreal Chickadee samples using four *P*_crit_ thresholds.

*N*_e_ estimates using NeEstimator had relatively broad confidence intervals for all three populations of interest (Table A6 in Appendix A). The historical Gray‐headed Chickadee *N*_e_ estimate was consistent as the *P*_crit_ threshold increased from 0 to 0.09, while the historical and contemporary western Boreal Chickadee *N*_e_ estimates increased with greater *P*_crit_ values (Figure 7). Contemporary western Boreal Chickadees had the greatest estimated *N*_e_ across all *P*_crit_ thresholds, and historical Gray‐headed Chickadees had the lowest estimated *N*_e_ values.

## 4 Discussion

As an endemic subspecies inhabiting a small geographic distribution, there is an urgent need to investigate the drivers of North American Gray‐headed Chickadee population declines, including hybridization with congeners. Due to the difficulty of locating and sampling Gray‐headed Chickadees, we used museum samples alongside contemporary samples of the widespread Boreal Chickadee to identify the first example of hybrid individuals involving this subspecies. Two Boreal Chickadees collected approximately 950 km apart in 1957 and 1961 were identified as first‐generation backcross hybrids, indicating the production of fertile hybrids in this system. We found additional signatures of Gray‐headed Chickadee nuclear loci introgressed into Boreal Chickadee genomes within both historical and contemporary individuals, and obtained genetic evidence (i.e., increase in effective population size over time) to support previous suggestions that Boreal Chickadees transitioned from being relatively uncommon to abundant in areas of sympatry. We also observed genetic structure between Gray‐headed Chickadees from North America and Eurasia, although this pattern may also be explained by temporal differences between sampling periods and associated genetic drift.

Many chickadee hybridization studies are designed to sample a gradient of hybrid indices across a contact zone, and thus do not provide a point of comparison for the frequency of hybrids observed (e.g., Wagner et al. 2020). One recent broad‐scale study using ddRAD data from a total of 400 Boreal Chickadees, Chestnut‐backed Chickadees, Black‐capped Chickadees, and Mountain Chickadees sampled throughout their ranges found seven early‐generation hybrids and generally extreme high or low hybrid index values among the rest of the individuals (Lait et al. 2024). Another study using ddRAD markers and similar analysis methods identified relatively higher hybrid frequency among 375 sympatric Black‐capped Chickadees and Mountain Chickadees, with 46 F1, F2, or first‐generation backcross individuals, as well as 115 second‐generation backcrosses (Grabenstein et al. 2023). Our detection of two early‐generation (first‐ or second‐generation) hybrids in the historical sample of 23 Gray‐headed Chickadees and 38 Boreal Chickadees is intermediate in the frequency reported previously. Notably, no early‐generation hybrids were detected among our larger set of 77 contemporary sympatric Boreal Chickadees. The lack of recent‐generation hybrids among the contemporary samples may indicate that encounter rates between Boreal Chickadees and Gray‐headed Chickadees are much lower currently than during the historical time period, in line with field survey reports. Several contemporary Boreal Chickadees exhibit low individual admixture percentages suggestive of late‐generation hybrids (Table A4 in Appendix A). Hybridization events, therefore, did not cease to occur. Characterization of such Boreal Chickadees as late‐generation hybrids is unlikely to be a misinterpretation of ancient introgression, as similar patterns were not observed in Boreal Chickadees from allopatric areas of western North America. Further sampling of Boreal Chickadees in areas of sympatry would be beneficial to improve our understanding of spatial patterns of introgression.

### 4.1 Asymmetrical Introgression Toward Boreal Chickadees

The absence of Gray‐headed Chickadee mtDNA introgression in hybrids suggests that hybrid pairs in the historical time period may have more frequently consisted of a female Boreal Chickadee and a male Gray‐headed Chickadee. The pattern of bias in hybrid parental sex aligns with mate scarcity expectations for the period when Boreal Chickadees were less locally abundant; we expect female birds to be more selective with mates as a function of their greater investment in offspring, and thus a rare species female will be more likely to mate with a heterospecific when compared to a female of the abundant species (Hubbs 1955; Wirtz 1999). Given the increase in Boreal Chickadee local abundance over time, supported by field surveys and our nuDNA effective population size estimates, we might expect that historical hybrids tend to have Boreal Chickadee mtDNA, while contemporary hybrids tend to have Gray‐headed Chickadee mtDNA. Both first‐generation backcrosses involved a Boreal Chickadee parental and had Boreal Chickadee mtDNA, despite the greater abundance of Gray‐headed Chickadees at the time. Wirtz's (1999) mate scarcity hypothesis regarding the tendency for rare species to contribute female parentals to hybrid crosses is also relevant to backcrossing, and females of a rare species can be overrepresented in backcross pairings as well (Peters et al. 2017).

In addition to mate scarcity‐driven patterns, behavioral or ecological dynamics can promote asymmetrical introgression into one species. Distinguishing between mate scarcity and other possible drivers of asymmetrical introgression is informative to understand Gray‐headed Chickadee and Boreal Chickadee interactions, as factors other than mate scarcity may lead to consistent historical and contemporary patterns. Understanding factors that may lead to biases in which parent species is more likely to mate with heterospecifics or hybrids can be complex, as each parent species can exhibit distinct sex‐specific mating preferences and dispersal patterns, among other possibilities.

Although there is little known about mating preferences in both species, captive studies of other chickadee species have shown that some females will preferentially choose to pair with a heterospecific. For example, captive studies suggest that Black‐capped Chickadee females and Carolina Chickadee females prefer to associate with Carolina Chickadee males once they observe dominance interactions between a male of each species (Bronson et al. 2003). Given the higher frequency of male hybrids predicted by Haldane's rule for birds (reviewed in Schilthuizen et al. 2011), such mate preferences could lead to a lower proportion of backcrosses to the socially dominant species. Observations of wild interactions or captive trials with Eurasian Gray‐headed Chickadees would be required to assess the possibility that Gray‐headed Chickadee males may be dominant to Boreal Chickadee males. If the asymmetry in hybridization direction we observed is due to a preference for Gray‐headed Chickadee‐type males that persists today, there may still be a bias toward introgression into contemporary Boreal Chickadee populations.

### 4.2 Demographic History of Gray‐Headed Chickadees and Boreal Chickadees

Demographic modeling with dadi estimated a low level of symmetrical, continuous gene flow between Gray‐headed Chickadees and Boreal Chickadees. Dadi is not well‐suited for modeling recent hybridization events, so our two datasets with different temporal Boreal Chickadee samples that share the same demographic history were devised to identify modeling incongruences that may be due to recent population changes. Using contemporary Boreal Chickadee samples instead of historical Boreal Chickadee samples in the two‐population model increased the estimated size of Boreal Chickadee populations in the recent time period compared to Gray‐headed Chickadees, which may be an indication of elevated genetic diversity in the contemporary Boreal Chickadee samples.

Model fit and biological parameter estimates of the historical Boreal Chickadee model are supported by the consistency of divergence time estimates with previous studies using mitochondrial divergence rates (Gill et al. 2005; Johansson et al. 2018), although the contemporary Boreal Chickadee sample model yielded a more recent split estimate (Figure 6). Researchers have posited that Gray‐headed Chickadees in North America may have been isolated in different glacial refugia or may have colonized North America after glacial retreat (DeCicco et al. 2017). Similar effective population size estimates for both species following divergence provide some support that Gray‐headed Chickadees in North America may have been in contact with eastern Siberian forms within the Beringian refugium during the Last Glacial Maximum. However, greater sampling within Russia is needed to confirm our hypothesis.

**FIGURE A3:**
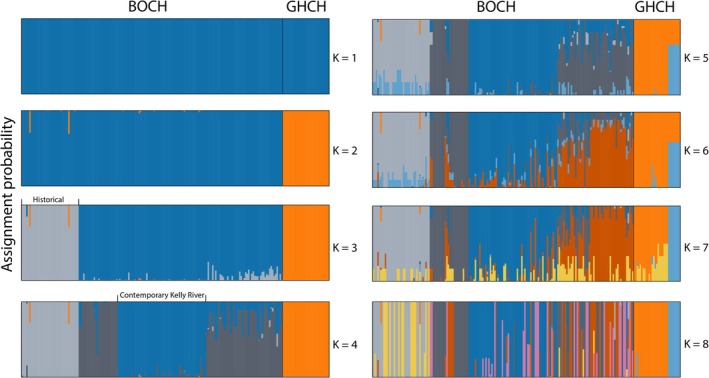
Assignment probabilities for *K* 1–8 based on ADMIXTURE analysis. Each column represents a single individual with species labels for Boreal Chickadee (BOCH) and Gray‐headed Chickadee (GHCH) listed.

Our hypotheses that sympatric Boreal Chickadee effective population size increased since the historical time period and that there has been gene flow between Nearctic and Palearctic Gray‐headed Chickadee subspecies post‐divergence are consistent with patterns of genetic structure uncovered by the results of the ADMIXTURE analysis. Within Boreal Chickadees, temporal structure between the historical and contemporary samples was recovered at *K* = 3 (Figure 2B), prior to observation of clustering within Gray‐headed Chickadee by subspecies (*K* = 5) or further clustering within Boreal Chickadee by region (*K* = 4; Figure A3 in Appendix A). The temporal shifts in allele frequencies may be attributable, at least in part, to genetic drift in small historical Boreal Chickadee populations on the western edge of their range which led to distinct genetic patterns, followed by immigration coupled with introgression and subsequent influx of genetic diversity.

### 4.3 Conservation Implications

Potential conservation actions to assist North American Gray‐headed Chickadees are challenged by their remote range, an inability to find individuals or populations, and our limited understanding of causes of decline. Although linking hybridization to population declines can be difficult, our findings indicate that hybridization has occurred and its genomic signature has been retained within both historical and contemporary Boreal Chickadees. Although hybridization has not previously been investigated or observed between Gray‐headed Chickadees and Boreal Chickadees, it was not unexpected given the records of hybrid offspring from crosses between the more distantly related Boreal Chickadees and Black‐capped Chickadees, as well as crosses between Eurasian Gray‐headed Chickadees and Willow Tits ( *P. montanus* ; Järvinen 1997; Lait et al. 2012). The lack of early‐generation hybrids detected in contemporary sympatric Boreal Chickadees despite sample sizes being greater than historical Boreal Chickadee and Gray‐headed Chickadee samples reinforces the potential severity of Gray‐headed Chickadee declines. Given the occurrence of hybridization as early as the 1960's in far western and eastern portions of the Gray‐headed Chickadee range, we do not find support for prioritizing a specific location as a better candidate for contemporary Gray‐headed Chickadee presence or as a refuge of genetic integrity.

Evidence of hybridization suggests that extant populations of Gray‐headed Chickadees likely contain introgressed alleles from Boreal Chickadees. Therefore, evaluating signatures of introgression for successfully captured Gray‐headed Chickadees would be germane to conservation plans, notably translocation and captive breeding efforts, to assess the genetic integrity of contemporary Gray‐headed Chickadee individuals. Further, the inclusion of genetic assessments to any future monitoring efforts of Boreal Chickadees may serve as a method to prioritize areas for intensive Gray‐headed Chickadee surveys. Areas with the greatest signals of Gray‐headed Chickadee introgression (e.g., early‐generation hybrids) may be potential areas of Gray‐headed Chickadee occupancy, given our findings of generally low levels of introgression within contemporary Boreal Chickadees.

Although hybridization is not uncommon among chickadees that co‐occur, the detection of introgression between Gray‐headed Chickadees and Boreal Chickadees has important implications regarding both the genetic and species integrity of Gray‐headed Chickadees. Specifically, the historical and contemporary range of Gray‐headed Chickadees largely overlaps with the more widespread Boreal Chickadee with very restricted areas of allopatry. Based on historical distribution patterns, Gray‐headed Chickadees are rare across much of their range with only small areas of higher abundance. This pattern of local abundance and restricted distribution of one species paired with the widespread abundance of the second species differs from instances of other hybridizing chickadee species. Many hybridizing species have a well‐defined hybrid zone (e.g., Black‐capped Chickadees and Carolina Chickadees; Taylor et al. 2014) accompanied by backcrossing into “pure” parental areas that aid in maintaining species integrity. Within our study system, there is likely limited opportunity for immigration or gene flow from “pure” populations of Gray‐headed Chickadees to mitigate the potential negative effects of genetic swamping or demographic swamping. Given the scarcity of Gray‐headed Chickadees and the extent of range overlap with Boreal Chickadees, the likelihood of genetic erosion due to ongoing hybridization and decreased population growth rates is a conservation concern for Gray‐headed Chickadees in North America.

Historical specimens and survey records have significantly contributed to our understanding of Gray‐headed Chickadees (Booms et al. 2020). Genetic material from samples that were collected before declines became apparent has supplied inferences about the current status and trajectory of Gray‐headed Chickadees, affirming the importance of proactively collecting genetic samples so that populations are archived before conditions change. Particularly important are samples from northern latitudes, where large environmental changes might lead to species distribution shifts and altered species interactions (Blois et al. 2013; Van Beest et al. 2021). The pairing of historical and contemporary samples in this study has afforded us the opportunity to explore interspecies interactions involving a rare species that has not been detected in nearly a decade. Opportunistic collection of genetic samples from both rare and common sources can greatly enhance our understanding of species response to climate‐mediated introgression among other Boreal and Arctic breeding species and might offer opportunities to inform active management and genetic rescue efforts.

## Author Contributions

**Matthew R. Armstrong:** formal analysis (lead), investigation (equal), writing – original draft (lead). **Robert E. Wilson:** data curation (equal), formal analysis (supporting), investigation (equal), writing – original draft (supporting). **James A. Johnson:** conceptualization (equal), funding acquisition (equal), project administration (equal), resources (equal), writing – review and editing (equal). **Travis L. Booms:** conceptualization (equal), writing – review and editing (equal). **Callie F. Gesmundo:** resources (supporting), writing – review and editing (equal). **Zachary M. Pohlen:** resources (supporting), writing – review and editing (equal). **Paul B. Leonard:** resources (supporting), writing – review and editing (equal). **Sarah A. Sonsthagen:** conceptualization (equal), data curation (equal), formal analysis (supporting), funding acquisition (equal), investigation (equal), project administration (equal), resources (equal), writing – original draft (supporting).

## Conflicts of Interest

The authors declare no conflicts of interest.

## Data Availability

Sample information and associated accession numbers are available at Sonsthagen et al. (2025). Raw data reads and mitochondrial control region sequences are available on NCBI Sequence Read Archive (BioProject PRJNA1236661, Biosample accessions: SAMN47401514–SAMN47401719) and GenBank (Accession Numbers: PV610848–PV611052).

## Appendix A

Text A1. Conversion of dadi model parameters to biologically relevant values was performed with the following formulas (model parameters bolded) [SNP = single nucleotide polymorphism]:

$$N_{\text{ancestral}}=\frac{\theta }{4*\text{yearly mutation rate}*\text{generation time}*L}$$

$$L\;\left(\text{Length of sequence}\right)\approx \text{base pairs after quality filtration}*\frac{\text{Segregating site count after projection}}{\mathrm{SNP}\;\text{count prior to thinning}}$$

$${\text{Time}}_{i}\;\left(\text{in years}\right)=2*T_{i}*N_{\text{ancestral}}*\text{generation time}$$

$$\mathrm{mig}\;\left(\text{Portion of population that}\;\mathrm{are}\;\mathrm{new}\;\text{migrants each generation}\right)=\frac{m}{2*N_{\text{ancestral}}}$$

**TABLE A1:** List of (A) Boreal Chickadee ( *Poecile hudsonicus* ) and (B) Gray‐headed Chickadee ( *P. cinctus* ) studied; including population assignment, sample time period, and sample source (MVZ = Museum of Vertebrate Zoology Bird Collection, University of California Berkeley; USNM = National Museum of Natural History Bird Collection, Smithsonian Institution; UAM = University of Alaska Museum of the North Bird Collection; UWBM = University of Washington Burke Museum Ornithology Collection; NYSM = New York State Museum Bird Collection).

(A)	
Kobuk River, Alaska, USA—Historical	MVZ38773, MVZ38774, MVZ38775, MVZ38776, MVZ38777, MVZ38778, MVZ38779, MVZ38780, MVZ38781, MVZ38782, MVZ38783, MVZ38784, MVZ38785
Kelly River, Alaska, USA—Historical	MVZ158358, MVZ158359, MVZ158360
Interior Alaska, USA—Historical	USNM435291, USNM435292, USNM466856, USNM466857, USNM466858, USNM 70832
Old Crow River, Yukon, Canada—Historical	USNM468107, USNM468108, USNM468109, USNM468110, USNM468111, USNM468112, USNM468113, USNM468114, USNM469307
Sheenjek and Coleen, Alaska, USA—Historical	UAM1054, UAM1061, UAM1064, UAM1492, UAM3757, UAM829, UAM876
Kelly River, Alaska, USA—Contemporary	58390, 58401, 58402, 58403, 58404, 58405, 58406, 58407, 58408, 58409, 58410, 58411, 58412, 58413, 58414, 58415, 58416, 58417, 58418, 58419, 58420, 58421, 58422, 58423, 58424, 58425, 58426, 58427, 58428, 58429, 58430, 58431, 58432, 58433, 58434, 58435, 58436, 58437, 58438, 58439, 58440, 58441, 58442, 58443, 58444, 58445, 58446, 58447, 58448, 58449, 58450, 58451, 58452, 58453, 58454, 58455, 58456, 58457, 58458
Sheenjek and Coleen, Alaska, USA—Contemporary	58675, 58676, 58677, 58678, 58679
Chicken, Alaska, USA—Contemporary	58467, 58468, 58469, 58470, 58471, 58472, 58473, 58474, 58475, 58476, 58477, 58478, 58479
Anchorage, Alaska, USA—Contemporary	7073, 7094, 7095, 7098, 7143, 7149, 7173, 7184, 7189, 7191, 7287, 10063, 10072, 10468, 10485, 10731, 10940, 10959, 10969, 10973, 10974, 11232, 11237, 11712, 71517
British Columbia, Canada—Contemporary	UWBM116188, UWBM116193, UWBM126660
Washington, USA—Contemporary	UWBM72378, UWBM72379, UWBM72380, UWBM72381, UWBM90245
Newfoundland, Canada—Contemporary	UWBM110766, UWBM110767, UWBM66783, UWBM78565, UWBM79510, UWBM79511
Maine, USA—Contemporary	NYSM15378, NYSM15380, NYSM15386, NYSM15387, NYSM15397, NYSM16650, NYSM16653, NYSM16663, NYSM16664, NYSM16665
New York, USA—Contemporary	NYSM11097, NYSM11108, NYSM11109, NYSM11187, NYSM11188, NYSM11189, NYSM11220, NYSM11221
Vermont, USA—Contemporary	NYSM14315, NYSM14316, NYSM14317

(B)	
Old Crow River, Yukon, Canada—Historical	USNM299322, USNM299323, USNM299324, USNM299325, USNM299326, USNM299327, USNM299328, USNM299329, USNM299331, USNM299332, USNM299333, USNM299351
Kobuk River, USA—Historical	MVZ38762, MVZ38763, MVZ38764, MVZ38765, MVZ38766, MVZ38767, MVZ38768, MVZ38769, MVZ38770, MVZ38771, MVZ38772
Russia—Contemporary	UWBM51874, UWBM51875, UWBM51881, UWBM78277, UWBM78419, UWBM78421, UWBM78430, UWBM78435

*Note:* Samples collected by U.S. Fish and Wildlife Service are referred to by U.S. Geological Survey aluminum band number without an institution code.

**TABLE A2:** Sequence information for primers used in mitochondrial control region amplification and sequencing.

Primer name	Sequence 5′–3′	Source
LmochCR1	CAGGGTATGYATGTCTTTGCATTC	Lait and Burg (2013)
BOCH_271H	GAGGGGAGTTTCCGATCTC	Designed for study
BOCH_237L	CCATTTAGCCCAACTGATCC	Designed for study
BOCH_406H	CTGGCTTGGGRGCTCCTG	Designed for study
BOCH_363L	GCATTCACGAAGTCCATAGC	Designed for study
BOCH_516H	TCGCGCTCGGGGTTTAGG	Designed for study
BOCH_513L	GGCTTCARGGACTTAAACTCC	Designed for study
BOCH_657H^a^	GTGAAGAGACGGTCGGATG	Designed for study
BOCH_707H	CTGCTACGCACTGGAAGG	Designed for study
BOCH_675L	CATCCGACCGTCTCTTCAC	Designed for study
BOCH_724L^a^	CCTTCCAGTGCGTAGCAG	Designed for study
H1015chCR	GCGGGTTTAACGAATGTGG	Lait and Burg (2013)

*Note:* In primer names, L denotes forward primers and H denotes reverse primers.

^a^ Sequencing only.

**TABLE A3:** Results of the analysis of molecular variance (AMOVA) from mitochondrial control region for populations grouped to assess partitioning of genetic variation in Boreal Chickadee (BOCH) and Gray‐headed Chickadee (GHCH) with among group (*Φ*_CT_), among populations within groups (*Φ*_SC_), within populations variance (*Φ*_ST_), and degrees of freedom (df) shown for each hierarchical analysis.

	Among groups		Among populations within groups		Within populations	
	*Φ*_CT_	df	*Φ*_SC_	df	*Φ*_ST_	df
Group 1 vs. 2 vs. 3 vs. 4 vs. 5	**0.874**	4	**0.076**	13	**0.883**	187
Historical and contemporary sympatric BOCH (Group 1 vs. 2)	−0.035	1	**0.085**	6	**0.052**	107
Sympatric and allopatric contemporary western BOCH (Group 2 vs. 3)	0.004	1	0.049	4	**0.053**	104
Allopatric contemporary western and eastern BOCH (Group 3 vs. 4)	**0.461**	1	0.024	5	**0.474**	53

*Note:* Populations ascribed to each group are listed in Table 1. Significant values are in bold text (*p* < 0.05).

**TABLE A4:** Hybrid index and hybrid class assignment probabilities for Boreal Chickadees (BOCH) with putative mixed ancestry based on ADMIXTURE analyses (*n* = 17).

Individual	Hybrid index	Hybrid class assignment	Posterior probability
BOCH‐38783‐KR‐H	0.0032	BOCH	1.00000
BOCH‐58436‐Kelly‐C	0.0043	BOCH	1.00000
BOCH‐58467‐CKN‐C	0.0044	BOCH	1.00000
BOCH‐58430‐Kelly‐C	0.0047	BOCH	1.00000
BOCH‐38782‐KR‐H	0.0048	BOCH	1.00000
BOCH‐38781‐KR‐H	0.0063	BOCH	1.00000
BOCH‐58407‐Kelly‐C	0.0063	BOCH	1.00000
BOCH‐70832‐InteriorAK‐H	0.009	BOCH	1.00000
BOCH‐58403‐Kelly‐C	0.0092	BOCH	1.00000
BOCH‐58424‐Kelly‐C	0.0098	BOCH	1.00000
BOCH‐468107‐OCR‐H	0.0102	BOCH	1.00000
BOCH‐58415‐Kelly‐C	0.014	BOCH	1.00000
BOCH‐58444‐Kelly‐C	0.0159	BOCH	1.00000
BOCH‐58454‐Kelly‐C	0.0182	BOCH	1.00000
BOCH‐58414‐Kelly‐C	0.0341	BOCH	1.00000
BOCH‐158359‐Kelly‐H	0.2863	Backcross BOCH	1.00000
BOCH‐468112‐OCR‐H	0.3034	Backcross BOCH	1.00000

*Note:* The hybrid index metric is based on posterior probability of assignment to the pure Gray‐headed Chickadee (GHCH) class using *gghybrid*, while hybrid class assignment is the posterior probability of assignment to one of six classes in the *hybriddetective* analysis.

**TABLE A5:** Ranking of the best fitting replicate for each demographic model in dadi and associated parameters for (A) the historical Gray‐headed Chickadee (GHCH)—historical Boreal Chickadee (BOCH) dataset and (B) the historical GHCH—contemporary sympatric BOCH dataset.

(A) Historical GHCH and Historical BOCH				Optimized parameters											
Model	Log‐likelihood	AIC	theta	nu1	nu2	*T*	*m*	nu1a	nu2a	nu1b	nu2b	m12	m21	T1	T2
sym_mig_size	−799.86	1613.7	303.35	—	—	—	0.10	0.58	0.39	4.28	2.76	—	—	0.79	0.18
asym_mig_size	−816.84	1649.7	390.35	—	—	—	—	0.08	0.04	2.80	1.09	0.03	0.27	0.09	0.31
sec_contact_asym_mig_size	−887.57	1791.1	113.35	—	—	—	—	1.76	2.53	11.73	2.43	0.03	0.14	3.62	0.54
sec_contact_asym_mig	−1120.32	2252.6	151.04	4.35	1.35	—	—	—	—	—	—	0.02	0.10	0.66	1.92
sym_mig	−1165.58	2339.2	195.5	2.85	1.20	2.13	0.06	—	—	—	—	—	—	—	—
asym_mig	−1172.2	2354.4	301.54	1.90	0.64	1.13	—	—	—	—	—	0.03	0.19	—	—
no_mig_size	−1184.48	2381	297.42	—	—	—	—	1.25	0.42	6.08	2.75	—	—	0.50	0.11
no_mig	−1442.91	2891.8	289.52	2.30	0.79	0.78	—	—	—	—	—	—	—	—	—
sec_contact_sym_mig	−1729.15	3468.3	2843.2	0.11	0.06	—	2.63	—	—	—	—	—	—	2.72	8.83
sec_contact_sym_mig_size	−1729.15	3472.3	1926.1	—	—	—	1.78	7.06	0.25	0.16	0.08	—	—	2.74	13.20

(B) Historical GHCH and sympatric contemporary BOCH				Optimized parameters											
Model	Log‐likelihood	AIC	theta	nu1	nu2	*T*	*m*	nu1a	nu2a	nu1b	nu2b	m12	m21	T1	T2
sym_mig_size	−879.42	1772.8	409.04	—	—	—	0.12	0.42	0.23	2.34	3.56	—	—	0.40	0.11
sec_contact_asym_mig_size	−1007.24	2030.5	209.24	—	—	—	—	1.65	1.21	3.73	1.03	0.06	0.20	2.02	0.17
asym_mig_size	−1080.62	2177.2	229.57	—	—	—	—	2.98	0.08	2.51	1.33	0.03	0.12	0.47	0.85
sec_contact_sym_mig	−1123.02	2256	204.8	2.58	1.13	—	0.12	—	—	—	—	—	—	1.95	0.15
sec_contact_sym_mig_size	−1151.74	2317.5	139.15	—	—	—	0.03	1.63	0.46	3.53	2.08	—	—	0.27	3.30
sym_mig	−1158.5	2325	100.01	5.19	2.56	5.23	0.03	—	—	—	—	—	—	—	—
asym_mig	−1198.06	2406.1	234.63	2.22	1.04	1.68	—	—	—	—	—	0.02	0.13	—	—
no_mig_size	−1330.9	2673.8	279.27	—	—	—	—	1.34	0.26	2.47	2.34	—	—	0.25	0.28
no_mig	−1480.06	2966.1	280.19	2.04	0.91	0.92	—	—	—	—	—	—	—	—	—
sec_contact_asym_mig	−1571.47	3154.9	2949.7	0.13	0.05	—	—	—	—	—	—	0.49	5.62	0.10	9.80

*Note:* The top model based on log‐likelihood for both datasets was symmetrical migration with an instantaneous size change. Model names incorporate the following abbreviations: No_mig = no migration, sym_mig = symmetrical migration, asym_mig = asymmetrical migration, size = instantaneous size change, and sec_contact = secondary contact. Optimized parameter values are rounded to two decimal places, and hyphens denote unused parameters for a given model. Models were selected from those published in the dadi_pipeline tool (Portik et al. 2017).

**TABLE A6:** Effective population size (*N*_e_) estimates for contemporary western Boreal Chickadees (BOCH), historical BOCH, and historical Gray‐headed Chickadee (GHCH) populations.

	*P*_crit_			
	0	0.03	0.06	0.09
Contemporary BOCH—Western				
*N*_e_ estimate	1683.4	2003.8	2045.6	2283.5
Lower bound	1202.8	1157.6	1044.5	1014.8
Upper bound	4671	8246.1	9378.9	∞
Historical BOCH				
*N*_e_ estimate	80.2	304.2	459.1	601.5
Lower bound	48.4	119.7	139.3	165.1
Upper bound	116.9	∞	∞	∞
Historical GHCH				
*N*_e_ estimate	15.7	109	78.7	69.7
Lower bound	6.2	60.1	41.3	38.2
Upper bound	68.5	557.6	423.6	268.1

*Note:* Estimates are displayed for three different minimum allele frequency (*P*_crit_) values, with upper and lower bounds based on jackknife confidence intervals. Upper confidence intervals of infinity are denoted with an ∞ symbol.
